# Supplementary material for: Serum interferon-gamma-induced protein 10 levels can help predict sarcopenia development in patients with primary hepatocellular carcinoma: A retrospective cohort study
Source: PLoS One. 2025 May 15;20(5):e0309852. doi: 10.1371/journal.pone.0309852 (PMC12080777; doi:10.1371/journal.pone.0309852)
Supplement: Attachment — Submitted filename: Response to Reviewers.docx [file pone.0309852.s002.docx]

Dear Dr. Amir Radfar,

Academic Editor

and the reviewers, PLOS ONE

PONE-D-24-35270

Serum interferon-gamma-induced protein 10 levels can help predict sarcopenia development in patients with primary hepatocellular carcinoma: A retrospective cohort study

Thank you for your kind review of our manuscript.

We appreciate receiving this opportunity to re-submit our manuscript.

Considering the suggestions, we have written Point by Points. We believe that these changes will alleviate the concerns of the reviewers. We would greatly appreciate if our manuscript could be accepted for publication in PLOS ONE.

Sincerely,

Hitomi Takada, MD.

Gastroenterology and Hepatology Department of Internal Medicine, Faculty of Medicine,

University of Yamanashi,

Yamanashi, Japan

**Point by points**

- **Reviewer 1**

1. **The introduction or discussion could possibly add more context to the role of IP10 in inflammation and tumorigenesis [1]. As there is existing research that suggests that IP10 modulates the tumour microenvironment of fibrosis associated HCC [2] [3] , as well as higher levels of inflammatory markers in age-related sarcopenia [4], I would be interested to know the association between disease progression with IP10 levels and sarcopenia in this context as I wonder whether the levels of IP10 actually reflect the levels of the pro-inflammatory environment and affect tumour modulation as well as the development of sarcopenia.**

Thank you very much for your detailed comments. As you pointed out, the relationship between IP-10 levels, levels of the pro-inflammatory environment and tumor modulation was not adequately described in our manuscript.

Thank you also for the important references.

We have modified the text as follows.

(line 235, page 21)

Aging is also associated with a chronic state of increased plasma levels of pro-inflammatory mediators, such as tumor necrosis factor α (TNFα), interleukin 6 (IL-6) and C-reactive protein (CRP), hence IP-10 may reflect levels in the inflammatory environment [26].

(line 257, page 22)

Deletion of IP-10 in fibrosis-associated HCC mice leads to the enhancement of anti-tumoral immune cells and an overall reduction of chemokines, but to a specific accumulation of chemokines at the tumor site [31, 32]. Thus, IP-10 levels have also been reported to modulate the tumor microenvironment of fibrosis-associated HCC. In this study, more patients with high baseline IP-10 levels or 1-year ratios showed recurrence beyond up to seven criteria during the follow-up period. IP-10 levels may be associated not only with the development of sarcopenia but also with tumor modulation.

2. **The authors have referred to some breadth of literature regarding how IP10 levels are correlated with sarcopenia and I am curious whether the strength of this correlation is different in people with HCC compared to other groups of patients exhibiting sarcopenia. Is there an aetiological inflammation and sarcopenia as well as inflammation and tumorigenesis on the liver tissue and does IP10 have a role in both these pathways? I understand if the discussion may be outside the scope of the paper, however I wonder whether the authors think this finding has shown a role for future research into this central question.**

Thank you very much for your comments. Reasons suggesting that the association between IP-10 levels, sarcopenia and tumorigenesis may be stronger than in patients with other diseases are that systemic inflammation and oxidative stress-related signaling pathways are important not only in the development of sarcopenia, but also in fibrosis progression and HCC.

We have modified the text as follows.

(line 275, page 23)

Sarcopenia is associated with low grade systemic inflammation, as indicated by increased inflammatory cytokines, leading to oxidative stress. Moreover, inflammation and stress-related signaling pathways are important in the progression of fibrosis and HCC development, and may have been associated with the present results [36, 37]. IP-10 levels in HCC patients are associated with inflammation and tumor formation in liver tissue, suggesting that the association with the development of sarcopenia may be more robust than in patients with other diseases.

3. **In the methods section it is unclear to me what the original clinical indication was to measure the IP10 levels of the study group initially - was this routine, random or was it measured in a specific subgroup of patients? The authors acknowledge that there may be some selection bias and I believe answering this question will help the audience understand the extent to which such bias may exist. I would also like to know how these 120 people equally distributed in between the three groups were then selected from the base cohort. Was there any specific randomisation algorithm or was it entirely up to the researcher's judgement?**

Thank you very much for your comments. As you pointed out, our description was inappropriate.

We have modified the text as follows.

(line 83, page 5)

From a total of 738 patients at our hospital with a confirmed diagnosis of primary HCC from January 2008 to January 2021, 239 patients with Barcelona Clinic Liver Cancer (BCLC) stage A who had satisfactory imaging at baseline and were aged ≥20 years were selected, whereas those without sufficient blood samples for IP-10 assay, those with missing data, those diagnosed with HCC other than BCLC stage A, or those with a shorter follow-up (<3 years) were excluded from the analysis. The presence of sarcopenia was further assessed at baseline and after 3 years, and patients were classified into the following three groups; patients with sarcopenia at baseline were classified as the Sarco-base group, patients who met the criteria for sarcopenia for the first time at 3-year follow-up were classified as the Sarco-develop group and patients who never met the criteria at follow-up were classified as the Non-Sarco group. In each group, 40 patients were selected from those with a newer date of first visit, and total 120 patients whose serum IP-10 levels were measured both at baseline and after 1 year, were enrolled in our research.

4. **It is interesting to note that the opposing effects of IP10 on muscle regeneration seen in previous studies has been encountered again and that too in the same patient population with different effects on muscle mass at diagnosis and at follow up after treatment has been initiated. Does this imply that the mechanism by which IP10 influences muscle mass changes with disease progression or with treatment.**

Thank you very much for your detailed comments. As you pointed out, the possibility that disease progression and treatment interventions may alter the relationship between IP-10 levels and the development of sarcopenia is an interesting point.

We have modified the text as follows.

(line 296, page 24)

In addition, in this study, among the group with high baseline IP-10 levels or 1-year ratios, patients who had undergone TACE more than twice during the follow-up period developed sarcopenia more frequently than those who had undergone TACE less than twice (86 vs. 50%, p=0.006). This suggests that disease progression and therapeutic interventions may influence the mechanism by which IP-10 influences muscle mass.

- **Reviewer 2**

1. **IP-10 values are influenced by many factors, as is recognized. So, although the statistical association exists in this group of patients, it seems too early to adopt the IP-10 level for intervention in individual patients. It is essential to study this association prospectively and include other factor which should be considered important as potential prognostic factors for sarcopenia.**

Thank you very much for your detailed comments. As you pointed out, it is premature to relate the association between the development of sarcopenia and IP-10 levels in this study alone. Prospective studies including other risk factors for the development of sarcopenia, are warranted in future.

(line 308, page 24)

Moreover, IP-10 levels are an item influenced by many factors and it is premature to relate the association between the development of sarcopenia and IP-10 levels in this study alone. Further prospective studies of this association, including other risk factors for the development of sarcopenia, are warranted.
