# Supplementary material for: Serum interferon-gamma-induced protein 10 levels can help predict sarcopenia development in patients with primary hepatocellular carcinoma: A retrospective cohort study
Source: PLoS One. 2025 May 15;20(5):e0309852. doi: 10.1371/journal.pone.0309852 (PMC12080777; doi:10.1371/journal.pone.0309852)
Supplement: Attachment — Submitted filename: Response to Reviewers v2.docx [file pone.0309852.s003.docx]

Dear Dr. Amir Radfar,

Academic Editor

and the reviewers, PLOS ONE

PONE-D-24-35270

Serum interferon-gamma-induced protein 10 levels can help predict sarcopenia development in patients with primary hepatocellular carcinoma: A retrospective cohort study

Thank you for your kind review of our manuscript.

We appreciate receiving this opportunity to re-submit our manuscript.

Considering the suggestions, we have written Point by Points. We believe that these changes will alleviate the concerns of the reviewers. We would greatly appreciate if our manuscript could be accepted for publication in PLOS ONE.

Sincerely,

Hitomi Takada, MD.

Gastroenterology and Hepatology Department of Internal Medicine, Faculty of Medicine,

University of Yamanashi,

Yamanashi, Japan

**Point by points**

- **Editor**

1- **Please provide a detailed point-by-point response to the reviewers in the re-submission package, explaining how each concern was addressed.**

Thank you for your kind guidance.

We will describe the amendments in the form of point by point.

2- **Please include precise** **details on patient inclusion and exclusion criteria, clarifying the rationale for the chosen patient groups and the term 'newer date of first visit'.**

Thank you very much for your detailed comments. As you pointed out, the details on patient inclusion and exclusion criteria were not fully explained in the original manuscript. Moreover, the term ‘newer date of first visit’ was used to refer to patients whose first visit date was closer to the present, i.e. with a short serum sample cryopreservation period. However, the statement was not stated with enough consideration. We have modified the text as follows.

(line 91, page 4)

In each group, 40 patients whose first visit date were closer to the present, i.e. with a short serum sample cryopreservation period were selected, and total 120 patients whose serum IP-10 levels were measured both at baseline and after 1 year, were enrolled in our research.

3- **Please propose a revised table structure that reduces redundancy while ensuring critical data is not omitted.**

Thank you very much for your detailed comments. As you pointed out, the tables in the original manuscript were redundant and difficult to read. We have merged Tables 1 and 4, deleted Table 3 and revised them for easier readability. Moreover, we have corrected the statement regarding the table numbers in the text.

(line 183, page 12)

The outcomes of the comparative analysis of the patient characteristics among the three groups are summarized in Table 1.

(line 201, page 16)

Patients without sarcopenia at baseline but who had high IP-10 levels at baseline or IP-10 ratios after 1 year exhibited several distinguishing characteristics, which were as follows: high ALBI grade, low BCAA, low BTR, low platelet counts, more patients opting for TACE as treatment for primary HCC, and recurrence beyond up to seven criteria during the 3-year follow-up period (Table 4).

4- **Please clarify which data support the statement on T-cell dysregulation. If this is based on indirect evidence, acknowledge the limitation explicitly. If additional data (e.g., T-cell subsets, cytokine levels) are available, include them to substantiate the argument.**

Thank you very much for your detailed comments. As you pointed out, we have described an association of IP-10 levels and dysregulation of T-cell response. However, as this is an assumption based on indirect evidence and not the results obtained in this study, the description has been amended as follows.

(line 287, page 22)

Our findings, taken together with previous reports, may suggest that high IP-10 ratios in BCLC stage A patients may be associated with the development of sarcopenia via dysregulation of T-cell transfer to muscle, T-cell differentiation and receptor abnormalities [40]. Further validation of this assumption, e.g. by measuring T-cell subsets, is needed in the future.

5- **Please include detailed multivariable analysis and provide an interpretation of how confounders impact the results.**

Thank you very much for your detailed comments. As you point out, we should have presented the results of multivariate analysis on sarcopenia development. We have added Table.3, and modified the text as follows.

(line 192, page 13)

Independently related factors for sarcopenia development were age >68 years old, male, body mass index <24, with diabetes mellitus, high IP-10 levels at baseline, and high IP-10 ratios at 1 year (Table 3).

- **Reviewer #2**

Thank you for your kind comments.

- **Reviewer #3**

1- **In the context of development of sarcopenia authors use “at 3 years” and “during 3 years” interchangeable throught the script. Doing the analysis “at 3 years” would minimize immortal time bias. Examples: line 33, 91, 148, …**

Thank you for your kind guidance. As you pointed out, our description was inappropriate. This study only included cases that had been followed for more than three years, and the presence of sarcopenia development was determined by imaging after three years, which we have believed to some extent to have taken a considered approach to immortality bias. We have modified the text as follows.

(line 31, page 2)

Patients who had sarcopenia at baseline computed tomography imaging were assigned to the Sarco-base group, whereas those in whom sarcopenia was found for the first time after 3 years were assigned to the Sarco-develop group.

(line 42, page 2)

Contrarily, the group without sarcopenia at baseline and with high baseline IP-10 levels and IP-10 ratios at 1 year were more likely to develop sarcopenia after 3 years.

(line 87, page 4)

The presence of sarcopenia was further assessed at baseline and after 3 years, and patients were classified into the following three groups; patients with sarcopenia at baseline were classified as the Sarco-base group, patients who met the criteria for sarcopenia after 3 years were classified as the Sarco-develop group and patients who never met the criteria at follow-up were classified as the Non-Sarco group.

(line 146, page 6)

Forty patients did not experience sarcopenia (Non-Sarco group), 40 had sarcopenia after 3 years (Sarco-develop group), and 40 patients were assigned to the Sarco-base group.

(line 316, page 23)

Conversely, those not presenting with sarcopenia at the first occurrence of HCC and subsequently developed sarcopenia after 3 years had higher baseline IP-10 levels and IP-10 ratios at 1 year.

2- **For these finding to be extrapolated to other populations patient selection has to be described in a more transparent fashion (line 92-95). “Newer date of first visit” has to be specified. Why exactly (these) 40 patients in each group?**

Thank you very much for your detailed comments. As you pointed out, our description about patient selection was inadequate in the original manuscript. Moreover, the term ‘newer date of first visit’ was used to refer to patients whose first visit date was closer to the present, i.e. with a short serum sample cryopreservation period. However, the statement was not stated with enough consideration. We have modified the text as follows.

(line 91, page 4)

In each group, 40 patients whose first visit date were closer to the present, i.e. with a short serum sample cryopreservation period were selected, and total 120 patients whose serum IP-10 levels were measured both at baseline and after 1 year, were enrolled in our research.

3- **There are 5 tables presenting aggregated patient data. Could some tables be joined together? Also, in Table 4 IP-10 levels at 1y next to baseline levels could be informative.**

Thank you very much for your detailed comments. As you pointed out, the tables in the original manuscript were redundant and difficult to read. We have merged Tables 1 and 4, deleted Table 3 and revised them for easier readability. We have modified Table 1 (Table 4 in the original manuscript), and the statement regarding the table numbers in the text.

(line 183, page 12)

The outcomes of the comparative analysis of the patient characteristics among the three groups are summarized in Table 1.

(line 201, page 16)

Patients without sarcopenia at baseline but who had high IP-10 levels at baseline or IP-10 ratios after 1 year exhibited several distinguishing characteristics, which were as follows: high ALBI grade, low BCAA, low BTR, low platelet counts, more patients opting for TACE as treatment for primary HCC, and recurrence beyond up to seven criteria during the 3-year follow-up period (Table 4).

4- **In the discussion section the authors make a statement of an association of IP-10 levels and dysregulation of T-cell response. What result from your analysis support this statement? Are there any further data not provided (measurements of T-cell subsets, cytokines,)?**

Thank you very much for your detailed comments. As you pointed out, we have described an association of IP-10 levels and dysregulation of T-cell response. However, as this is an assumption based on indirect evidence and not the results obtained in this study, the description has been amended as follows.

(line 287, page 22)

Our findings, taken together with previous reports, may suggest that high IP-10 ratios in BCLC stage A patients may be associated with the development of sarcopenia via dysregulation of T-cell transfer to muscle, T-cell differentiation and receptor abnormalities [40]. Further validation of this assumption, e.g. by measuring T-cell subsets, is needed in the future.

- **Reviewer #4**

1- **Abstract. The results sound confusing. “baseline IP-10 … significantly lower in the Sarco-base group compared to the rest”, but it further states that “the baseline IP-10 levels were higher in the Sarco-develop group than in the Sarco-base…”.**

Thank you very much for your detailed comments. As you pointed out, our description in abstract section was inadequate in the original manuscript. We have modified the text as follows.

(line 37, page 2)

Conversely baseline IP-10 levels and IP-10 ratio at 1 year were higher in the Sarco-develop group than in the Non-Sarco group (p = 0.0017, p = 0.025).

High IP-10 levels at baseline, and high IP-10 ratios at 1 year were independently related factors for sarcopenia development.

2- **Table 4. I assume that the comparison for Table 4 was conducted with baseline data. But please clarify this.**

Thank you very much for your detailed comments. As you pointed out, comparisons in Table 4 are made with baseline data. Characteristics of the three groups have been modified to present with baseline data in all cases in Table 1.

3- **It’s interesting to observe lower IP-10 levels at baseline for patients with sarcopenia but a higher chance of developing sarcopenia for those with higher IP-10 levels at baseline. As all the analyses were conducted between two variables without considering other covariates, was this opposite direction scenario due to a lack of consideration of other confounding variables when quantifying the relationship between IP-10 level and the status of sarcopenia? Multiple variable analysis should be conducted to clarify this.**

Thank you very much for your detailed comments. As you point out, we should have presented the results of multivariate analysis on sarcopenia development. We have added Table.3, and modified the text as follows.

(line 192, page 13)

Independently related factors for sarcopenia development were age >68 years old, male, body mass index <24, with diabetes mellitus, high IP-10 levels at baseline, and high IP-10 ratios at 1 year (Table 3).
