# Supplementary material for: Serum interferon-gamma-induced protein 10 levels can help predict sarcopenia development in patients with primary hepatocellular carcinoma: A retrospective cohort study
Source: PLoS One. 2025 May 15;20(5):e0309852. doi: 10.1371/journal.pone.0309852 (PMC12080777; doi:10.1371/journal.pone.0309852)
Supplement: Attachment — Submitted filename: Response to Reviewers v3.docx [file pone.0309852.s004.docx]

Dear Dr. Amir Radfar,

Academic Editor

and the reviewers, PLOS ONE

PONE-D-24-35270

Serum interferon-gamma-induced protein 10 levels can help predict sarcopenia development in patients with primary hepatocellular carcinoma: A retrospective cohort study

Thank you for your kind review of our manuscript.

We appreciate receiving this opportunity to re-submit our manuscript.

Considering the suggestions, we have written Point by Points. We believe that these changes will alleviate the concerns of the reviewers. We would greatly appreciate if our manuscript could be accepted for publication in PLOS ONE.

Sincerely,

Hitomi Takada, MD.

Gastroenterology and Hepatology Department of Internal Medicine, Faculty of Medicine,

University of Yamanashi,

Yamanashi, Japan

**Point by points**

- **Reviewer #5, Editor**

1. **Please describe all abbreviation as they appear in manuscript (e.g. ALBI, BCAA, BTR, BCLC) to be more understanadblke for readers not familiar with them**

Thank you for your kind guidance. The abbreviations you mentioned were used after the official name when first presented in the text of the manuscript. However, the text was rambling and may have been difficult to read for readers. The following are descriptions of the first occurrence of the abbreviations in the original manuscript. Please let us know again if any additional corrections are needed.

Albumin-bilirubin (ALBI) (line 156, page 11)

Branched-chain amino acids (BCAA) (line 157, page 11)

Branched-chain amino acids/tyrosine ratio (BTR) (line 157, page 11)

Barcelona Clinic Liver Cancer (BCLC) (line 83, page 4)

2. **Several times (lines 208, 220) you mentioned "high IP-10 value and IP-10 ratio" - please ensure is it high or low ratio level in order to be no doubt for the readers**

Thank you for your kind guidance. As you pointed out, our description was inappropriate. We have modified the text as follows.

Contrarily, the group without sarcopenia at baseline and with high baseline IP-10 levels and high IP-10 ratios at 1 year were more likely to develop sarcopenia after 3 years. (line 42, page 2)

Characteristics of patients with high baseline IP-10 levels or high ratios (line 201, page 16)

Patients without sarcopenia at baseline but who had high IP-10 levels at baseline or high IP-10 ratios after 1 year exhibited several distinguishing characteristics, which were as follows: high ALBI grade, low BCAA, low BTR, low platelet counts, more patients opting for transarterial chemoembolization (TACE) as treatment for primary HCC, and recurrence beyond up to seven criteria during the 3-year follow-up period (Table 4). (line 203, page 16)

Contrarily, in patients without sarcopenia at baseline, sarcopenia was more likely to develop in those with higher baseline IP-10 levels and higher 1-year IP-10 ratios. (line 214, page 19)

In this study, more patients with high baseline IP-10 levels or high 1-year ratios showed recurrence beyond up to seven criteria during the follow-up period. (line 256, page 20)

In addition, in this study, among the group with high baseline IP-10 levels or high 1-year ratios, patients who had undergone TACE more than twice during the follow-up period developed sarcopenia more frequently than those who had undergone TACE less than twice (86 vs. 50%, p=0.006). (line 294, page 22)

Conversely, those not presenting with sarcopenia at the first occurrence of HCC and subsequently developed sarcopenia after 3 years had higher baseline IP-10 levels and higher IP-10 ratios at 1 year. (line 325, page 24)

3. **Out of three groups equally divided by numbers which is interesting and not common in research, the Non-sarco group was the "youngest" one 65 Age Median, with BMI >24, and DM in >40% and with less male subjects than in other groups. Would you consider this as bias? Please explain and comment since you stated that age >68, BMI >24 and DM are independently related factor for sarcopenia development**

Thank you very much for your detailed comments. As you have pointed out, Non-Sarco group was younger and had fewer thin, diabetic and male subjects, and we believe that there is an effect of bias due to these covariates in the results of this study.　Therefore, propensity score matching was performed using these factors. After matching, age, BMI, DM, and gender did not remain as independent factors.　In contrast, high IP-10 levels at baseline and high IP-10 ratios at 1 year were still independently associated factors for the development of sarcopenia. We have modified the table 3 and text as follows.

In all the patients, independently related factors for sarcopenia development were age >68 years old, male, body mass index <24, with diabetes mellitus, high IP-10 levels at baseline, and high IP-10 ratios at 1 year (Table 3). After propensity score matching using the factors; age, BMI, DM, and gender, high IP-10 levels at baseline and high IP-10 ratios at 1 year were still independently associated factors for the development of sarcopenia (Table 3). (line 194, page 13)

The baseline IP-10 levels and the trends remained independent factors for the development of sarcopenia after matching. In contrast, the Non-Sarco group in the analysis of all the patients was younger, and had fewer thin, diabetic and male subjects, a bias that influenced the results of this study. Additional analyses in a large number of patients are needed on the reality of this situation and the importance of IP-10 levels using matching. (line 305, page 22)
